# Staphylococcus aureus Adapts to Oxidative Stress by Producing H_2_O_2_-Resistant Small-Colony Variants via the SOS Response

**DOI:** 10.1128/IAI.03016-14

**Published:** 2015-04-15

**Authors:** Kimberley L. Painter, Elizabeth Strange, Julian Parkhill, Kathleen B. Bamford, Darius Armstrong-James, Andrew M. Edwards

**Affiliations:** a MRC Centre for Molecular Bacteriology and Infection, Imperial College London, London, United Kingdom; b Wellcome Trust Sanger Institute, Wellcome Trust Genome Campus, Hinxton, Cambridge, United Kingdom; c Department of Microbiology, Hammersmith Campus, Imperial College Healthcare NHS Trust, London, United Kingdom; d Department of Medicine, Imperial College London, London, United Kingdom

## Abstract

The development of chronic and recurrent Staphylococcus aureus infections is associated with the emergence of slow-growing mutants known as small-colony variants (SCVs), which are highly tolerant of antibiotics and can survive inside host cells. However, the host and bacterial factors which underpin SCV emergence during infection are poorly understood. Here, we demonstrate that exposure of S. aureus to sublethal concentrations of H_2_O_2_ leads to a specific, dose-dependent increase in the population frequency of gentamicin-resistant SCVs. Time course analyses revealed that H_2_O_2_ exposure caused bacteriostasis in wild-type cells during which time SCVs appeared spontaneously within the S. aureus population. This occurred via a mutagenic DNA repair pathway that included DNA double-strand break repair proteins RexAB, recombinase A, and polymerase V. In addition to triggering SCV emergence by increasing the mutation rate, H_2_O_2_ also selected for the SCV phenotype, leading to increased phenotypic stability and further enhancing the size of the SCV subpopulation by reducing the rate of SCV reversion to the wild type. Subsequent analyses revealed that SCVs were significantly more resistant to the toxic effects of H_2_O_2_ than wild-type bacteria. With the exception of heme auxotrophs, gentamicin-resistant SCVs displayed greater catalase activity than wild-type bacteria, which contributed to their resistance to H_2_O_2_. Taken together, these data reveal a mechanism by which S. aureus adapts to oxidative stress via the production of a subpopulation of H_2_O_2_-resistant SCVs with enhanced catalase production.

## INTRODUCTION

*S*taphylococcus aureus is a frequent cause of chronic and recurrent infections, which often involve the emergence of slow-growing mutants known as small-colony variants (SCVs) (1–14).

The majority of SCVs isolated from clinical samples are auxotrophic for hemin, menadione, or thymidine due to mutations in the *hem* or *men* operons or in *thyA*, respectively (2, 4, 15–19). However, SCVs with mutations conferring resistance to fusidic acid or which arise via mutation in succinate dehydrogenase have also been identified, and there also appear to be isolates with a transient SCV phenotype, which are likely not mutants (12, 20, 21). SCVs with mutations in heme or menaquinone biosynthetic pathways have defective electron-transport chains, which confers resistance to aminoglycoside antibiotics such as gentamicin (1, 6, 8, 9).

Previous work has shown that gentamicin-resistant SCVs emerge in replicating populations in the absence of environmental stress via stochastic mutations but frequently revert to the wild type (WT) via the acquisition of suppressor mutations (15, 17, 22). However, while a few factors have been identified that select for the SCV phenotype, there is also evidence that environmental stimuli can trigger the emergence of SCVs in S. aureus populations, although the mechanism(s) by which this occurs is unknown (12, 23, 24).

In addition to aminoglycoside resistance, SCVs that arise via the loss of the electron transport chain are more tolerant than wild-type bacteria of other classes of bactericidal antibiotics (8, 9, 25–29). Furthermore, SCVs exhibit other phenotypic characteristics which may promote survival in host tissues, including elevated rates of host cell invasion and intracellular survival, enhanced capsule production, and robust biofilm formation (5, 12, 13, 30–32). Several of these phenotypes are ascribed to a combination of decreased Agr activity and enhanced SigB activity, which results in strong expression of surface proteins and an absence of cytolysin production (16, 33–35).

However, there is one aspect of the biology of electron-transport chain defective SCVs that appears to be at odds with a role in chronic infection: an apparently reduced level of defense against oxidative stress. This is important because the generation of reactive oxygen species (ROS) such as O_2_^−^ and H_2_O_2_ by neutrophils is a crucial host defense mechanism against S. aureus (36, 37). To combat ROS, S. aureus uses a number of defensive molecules, including catalase (KatA), superoxide dismutases (SodA/M), and the golden pigment staphyloxanthin (36–44). Despite the importance of these defenses for wild-type S. aureus survival in the host, SCVs have been reported to produce significantly reduced levels of staphyloxanthin and heme auxotrophs are deficient in catalase, which would be expected to make them more susceptible to ROS generated by neutrophils and thus clearance from host tissues (9, 19, 28, 36, 45). Therefore, the aim of this work was to determine the effect of ROS on the emergence and persistence of electron-transport chain defective SCVs within S. aureus populations and establish the degree to which SCVs are sensitive to oxidative stress.

## MATERIALS AND METHODS

### Bacterial strains and culture conditions.

Strains used in the present study are listed in Table 1. S. aureus was cultured in tryptic soy broth at 37°C with shaking as described previously (22). Broth cultures were inoculated with bacteria from stationary phase (10^5^ CFU ml^−1^), followed immediately by oxidants or ciprofloxacin and incubated for 16 h at 37°C with shaking at 180 rpm. Bacteria were used in stationary phase since this is when pigmentation of the wild type is greatest (36). Transposon mutants were cultured in the presence of erythromycin (10 μg ml^−1^), but subsequent assays were performed in the absence of the antibiotic to reduce off-target effects. CFU counts were determined by serial dilution and plating of aliquots onto tryptic soy agar (TSA) or Columbia blood agar (CBA) with or without gentamicin (2 μg ml^−1^). SCVs were defined as gentamicin-resistant (MIC > 2 μg ml^−1^) bacteria that produced small, slow-growing, nonhemolytic or weakly hemolytic, and nonpigmented or weakly pigmented colonies on blood agar. We did not study other types of SCVs, such as those resistant to sulfonamides or fusidic acid. DNA from transposon mutants was transduced into wild-type SH1000 by transduction with ϕ11 as described previously, and transductants bearing the inserted transposon were selected for on TSA containing erythromycin (10 μg ml^−1^) (46).

**TABLE 1:** Bacterial strains used in this study

Bacterial strain	Relevant characteristics^*a*^	Source or reference
E. coli		
DC10B	DNA cytosine methyltransferase deficient	50
DC10B/pCN34	DC10B transformed with pCN34	49; this study
DC10B/pCL55	DC10B transformed with pCL55	47
DC10B/p*hemB*	DC10B transformed with pCL55 containing the promoter of the *hem* operon fused to the coding sequence of *hemB*	
DC10B/p*menD*	DC10B transformed with pCL55 containing the promoter of the *men* operon fused to the coding sequence of *menD*	
DC10B/p*umuC*	DC10B transformed with pCN34 containing the promoter and coding region of *umuC*	
S. aureus		
SH1000	Functional *rsbU^+^* derivative of NCTC 8325-4	66
SCV2	SH1000-derived Gm^r^ SCV without auxotrophy for Men, Hem, Thy, CO_2_, or fatty acids; isolated in the absence of oxidants	This study
SCV4	SH1000-derived Gm^r^ SCV without auxotrophy for Men, Hem, Thy, CO_2_, or fatty acids; isolated in the absence of oxidants	
SCV9	SH1000-derived Gm^r^ SCV with auxotrophy for Hem; isolated in the absence of oxidants	
SCV13	SH1000-derived Gm^r^ SCV with auxotrophy for CO_2_; isolated in the absence of oxidants	
SCV14	SH1000-derived Gm^r^ SCV with auxotrophy for Men; isolated in the absence of oxidants; single nucleotide deletion in *menB*, leading to premature stop codon after 28 amino acids	
SCV15	SH1000-derived Gm^r^ SCV with auxotrophy for fatty acids; isolated in the absence of oxidants	
SCV17	SH1000-derived Gm^r^ SCV with auxotrophy for Men; isolated in the absence of oxidants	
SCV20	SH1000-derived Gm^r^ SCV with auxotrophy for CO_2_; isolated in the absence of oxidants	
SCV21	SH1000-derived Gm^r^ SCV without auxotrophy for Men, Hem, Thy, CO_2_, or fatty acids; isolated in the absence of oxidants	
SCV1036	SH1000-derived Gm^r^ SCV with auxotrophy for Men; isolated in the absence of oxidants	
SCV1045	Single nucleotide deletion in *menA*, leading to a premature stop codon after 184 amino acids	
SCV1047	SH1000-derived Gm^r^ SCV with auxotrophy for Men; isolated in the absence of oxidants; single nucleotide polymorphism in *menF*, resulting in A367D substitution of a highly conserved alanine	
SCV1057	SH1000-derived Gm^r^ SCV with auxotrophy for Men; isolated in the presence of H_2_O_2_; single nucleotide deletion in *menE*, leading to a premature stop codon after 205 amino acids	
SCV1058	SH1000-derived Gm^r^ SCV with auxotrophy for Men; isolated in the presence of H_2_O_2_; single nucleotide deletion in *menB*, leading to a premature stop codon after 113 amino acids	
SCV1060	SH1000-derived Gm^r^ SCV with auxotrophy for Men; isolated in the presence of H_2_O_2_; single nucleotide polymorphism in *menE*, leading to a premature stop codon after 165 amino acids	
SCV1072	SH1000-derived Gm^r^ SCV with auxotrophy for Men; isolated in the presence of paraquat; single nucleotide polymorphism in *menE*, leading toa premature stop codon after 373 amino acids	
SCV1072 *katA*::Tn	SCV1072 transduced with DNA from NE1366, resulting in inactivation of catalase; Ery^r^	
SCV1077	SH1000-derived Gm^r^ SCV with auxotrophy for Men; isolated in the presence of paraquat; single nucleotide polymorphism in *menF*, resulting in T79K substitution	
SCV1080	SH1000-derived Gm^r^ SCV with auxotrophy for Men; isolated in the presence of paraquat; single nucleotide polymorphism in *aroB*, resulting in H241Q substitution of a highly conserved histidine likely involved in metal binding	
SH1000t	SH1000-derived Tc^r^ strain *geh*::pTM304	22
MJH502	SH1000 *sigB*::Tc	66
SH331	SH1000 *rexA*::Tn	This study
SH445	SH1000 *umuC*::Tn	
SH805	SH1000 *recA*::Tn	
SH1012	SH1000 *rexB*::Tn	
SH1366	SH1000 transduced with DNA from NE1366, resulting in inactivation of catalase; Ery^r^	This study
SH1866	SH1000 *dinB*::Tn	
SH445/pCN34	SH1000 *umuC*::Tn transformed with pCN34	
SH445/p*umuC*	SH1000 *umuC*::Tn transformed with p*umuC*	
USA300 LAC	LAC strain of the USA300 CA-MRSA lineage	85
USA300 *hemB*	USA300 in which *hemB* has been deleted	35
USA300 *hemB geh*::pCL55	USA300 *hemB* mutant with pCL55 integrated into the *geh* locus	This study
USA300 *hemB geh*::p*hemB*	USA300 *hemB* mutant with p*hemB* integrated into the *geh* locus, restoring wild-type phenotype	
USA300 *menD*	USA300 in which *menD* has been deleted	35
USA300 *menD geh*::pCL55	USA300 *menD* mutant with pCL55 integrated into the *geh* locus	This study
USA300 *menD geh*::p*menD*	USA300 *menD* mutant with p*menD* integrated into the *geh* locus, restoring wild-type phenotype	
USA300 JE2	USA300 cured of plasmids	61
NE331	USA300 JE2 *rexA*::Tn	
NE445	USA300 JE2 *umuC*::Tn	
NE805	USA300 JE2 *recA*::Tn	
NE1012	USA300 JE2 *rexB*::Tn	
NE1366	USA300 JE2 *katA*::Tn	
NE1866	USA300 JE2 *dinB*::Tn	
CX003SCV	Clinical Men-auxotroph SCV	This study
CX003WT	Revertant of CX003SCV with wild-type phenotype	
CX004SCV	Clinical Men auxotroph SCV	
CX004WT	Revertant of CX004SCV with wild-type phenotype	
CX005SCV	Clinical Men auxotroph SCV	
CX005WT	Revertant of CX005SCV with wild-type phenotype	
CX006SCVM	Clinical Men auxotroph SCV	
CX006SCVH	Clinical Hem auxotroph SCV	
CX006WT	Revertant of CX005SCVM with wild-type phenotype	
CX009SCV	Clinical Hem auxotroph SCV	
CX009WT	Revertant of CX009SCV with wild-type phenotype	
Wood	Wild-type	NCTC 7121
MRSA252	Wild-type	86

a Gm^r^, gentamicin resistance; Tc^r^, tetracycline resistance; Ery^r^, erythromycin resistance; Hem, hemin; Men, menadione; Thy, thymidine.

USA300-derived *hemB* and *menD* mutants (35) were complemented by cloning the appropriate gene, under the control of the native promoter of the relevant operon, into integrative plasmid pCL55 (47). For *hemB*, the promoter region (48) was amplified by using the primer pair Hem Prom For (**CCTTTCGTCTTCAA**CGTATATTCATTGACCCG) and Hem Prom Rev (**GTCTATCAAATTTCAT**GTTCAATTCCTCCTAGG). For *menD*, the 331 bases upstream of *menF*, the first gene in the *men* operon, were amplified by using the primer pair Men Prom For (**CCCTTTCGTCTTCAA**TGAATACAAAACCTCTTTAAATC) and Men Prom Rev (**CTTTATGATTTCCCAT**ATAAAAGCGATCTCCTGCC). Amplicons containing promoter regions were fused with coding sequences by using the Gibson assembly protocol (NEB). DNA overhangs were built into primers (indicated in boldface) to facilitate recombination. The *hemB* gene was amplified by using the primer pair Hem For (**GGAGGAATTGAAC**ATGAAATTTGATAGACATAG) and Hem Rev (**TACCGAGCTCGAATTC**ACCTTAATTATCTAAATAGC), while *menD* was amplified by using the primer pair Men For (**GGAGATCGCTTTTAT**ATGGGAAATCATAAAGCAG) and Men Rev (**ACCGAGCTCGAATTC**TTATAATGTGTCATGAATCATTTC). The vector, pCL55, was amplified by using primers with overhangs to facilitate Gibson assembly. For *hemB* constructs, pCL55 was amplified by using the primer pair pCL55 Hem For (**AGATAATTAAGGT**GAATTCGAGCTCGGTACC) and pCL55 Hem Rev (**AATGAATATACG**TTGAAGACGAAAGGGCCTC), and for *menD* constructs, the primer pair pCL55 Men For (**CATGACACATTA**TAAGAATTCGAGCTCGGTAC) and pCL55 Men Rev (**AGAGGTTTTGTATTCA**TTGAAGACGAAAGGG) was used.

The SH1000 *umuC*::Tn mutant was complemented with the *umuC* coding sequence (including the promoter region) using pCN34 (49). The *umuC* gene and the promoter region was amplified by using the primer pair *umuC* For (AAAGGATCCCGGCGTCAGTTACTTCGC) and *umuC* Rev (AAAGGATCCCGTATCGCGACGCACTAC), which included BamHI restriction sites (underlined) to enable ligation into BamHI-digested pCN34. Vector without the *umuC* coding sequence served as a control. The successful generation of constructs was confirmed by DNA sequencing. Vectors were constructed in Escherichia coli strain DC10B and transformed directly into S. aureus strains (50). In the case of pCL55, plasmid integration was confirmed by PCR. DC10B was cultured in LB broth containing ampicillin (100 μg ml^−1^) where necessary to select for plasmid maintenance (50). S. aureus strains containing plasmids were cultured in the presence of 10 μg of chloramphenicol ml^−1^ (pCL55) or 90 μg of kanamycin ml^−1^ (pCN34) and washed in phosphate-buffered saline (PBS) to remove antibiotics, and experiments were performed in the absence of antibiotics to avoid off-target effects.

### Hydrogen peroxide quantification.

The concentration of H_2_O_2_ in culture medium was determined by using a Pierce quantitative peroxide assay kit according to the manufacturer's instructions.

### SCV stability assays.

The stability of SCV isolates was determined as described previously (22). SCV colonies (*n* = 30 to 50) on TSA plates containing 2 μg of gentamicin ml^−1^ were subcultured by streaking them onto antibiotic-free TSA using a sterile pipette tip, followed by incubation at 37°C for 48 h. Subsequently, subcultured bacteria were scored for reversion. If all colonies in the subcultured streak retained the SCV phenotype, then that SCV was scored as stable. If all of the colonies had the WT phenotype, the streak was scored as unstable. SCVs that generated a mixture of SCV and WT phenotype were categorized as partially stable (22).

### Phenotype-switching assay.

To understand the relative contributions of phenotype-switching and replication to determining the size of the SCV population, we used a previously described assay (22). Briefly, inocula of 10^5^ CFU tetracycline-sensitive wild-type SH1000 S. aureus and 10 CFU SH1000t tetracycline-resistant SCVs were cultured in the absence or presence of oxidants. The total CFU were quantified by plating serial dilutions on TSA plates. SCVs were isolated on TSA plates containing gentamicin, as described above. Subsequently, 100 SCV colonies were picked and patched onto TSA plates containing tetracycline to determine the percentage of SCVs that were resistant to the antibiotic. This assay determines the percentage of the final SCV population that arose from wild-type or SCV bacteria in the inoculum. Previous work has shown that the tetracycline-resistant strain does not suffer a fitness cost under the conditions used (22).

### SCV reversion assay.

Individual SCV colonies were picked from TSA plates containing gentamicin (2 μg ml^−1^) and resuspended in 150 μl of PBS. Aliquots (50 μl) of each bacterial suspension were then spread over TSA plates containing paraquat (0.1 mM), ciprofloxacin (0.05 μg ml^−1^), or neither before incubation for 24 h at 37°C. Subsequently, plates were examined for the presence of colonies of wild-type bacteria (large, pigmented colonies).

### Mutation rate analyses.

S. aureus strains were cultured in 3 ml of TSB after inoculation from agar plates. Cultures were diluted to 10^5^ CFU ml^−1^ in 30 parallel 1-ml cultures (this was the smallest inoculum that allowed bacterial growth in the presence of H_2_O_2_) and grown to stationary phase at 37°C with shaking. Total CFU counts were determined in 10 randomly selected cultures by plating of serial dilutions onto TSA without antibiotics. Each culture was then plated onto TSA containing rifampin (100 μg ml^−1^), followed by incubation for 24 h at 37°C. The number of resistant colonies was counted, and mutation rates with confidence intervals were calculated by using the maximum-likelihood setting of the FALCOR mutation rate calculator (51, 52). The statistical significances of differences between the mutation rate in the absence and presence of H_2_O_2_ were determined by using a Student *t* test as described in equation 5 of FALCOR (51, 52).

### Hydrogen peroxide killing assays.

S. aureus cells in late exponential phase (when pigmentation is strongest) were washed by sequential rounds of centrifugation and resuspension in PBS before subsequent adjustment to a final concentration of ∼10^6^ CFU ml^−1^ in PBS. Bacterial suspensions (10 μl) were added to the wells of a microtiter plate, and H_2_O_2_ was added to 30 mM for SH1000-derived isolates or 25 mM for USA300-derived strains (this concentration was chosen because preliminary assays indicated that they were the lowest required to achieve >1-log killing of the wild-type over 1 h [data not shown]). The microtiter plate was incubated at 37°C in the dark for 15 to 60 min. Surviving bacteria were enumerated by serial dilution in PBS and plating onto CBA (which naturally contains catalase to neutralize residual H_2_O_2_).

### Catalase activity assay.

S. aureus was grown and washed as described above for hydrogen peroxide killing assays before 10^7^ CFU were added to 1 ml of PBS containing 100 μM H_2_O_2_. The concentration of H_2_O_2_ was measured over time by using a Pierce quantitative peroxide assay kit in accordance with the manufacturer's instructions and the use of a standard plot.

### Whole-genome sequencing.

DNA was extracted from wild-type SH1000 and derived SCVs using lysostaphin and phenol-chloroform extraction (46). Purified DNA was sheared into fragments of ∼150 bp and sequenced using an Illumina MiSeq DNA sequencer. The sequences obtained yielded >100-fold coverage.

## RESULTS

### Culture of S. aureus in the presence of hydrogen peroxide leads to a specific and dose-dependent increase in the size of the SCV subpopulation.

To determine the effect of oxidative stress on the size of the gentamicin-resistant SCV subpopulation, S. aureus was cultured in the presence of increasing concentrations of H_2_O_2_, paraquat, or diamide, which have previously been shown to trigger distinct changes in the staphylococcal proteome (53).

We used an inoculum size (<10^5^ ml^−1^) that was predicted to not contain SCVs due to their low frequency in the population. Therefore, SCVs that appeared in the cultures were generated by the acquisition of mutations in wild-type cells (22).

**FIG 1:**
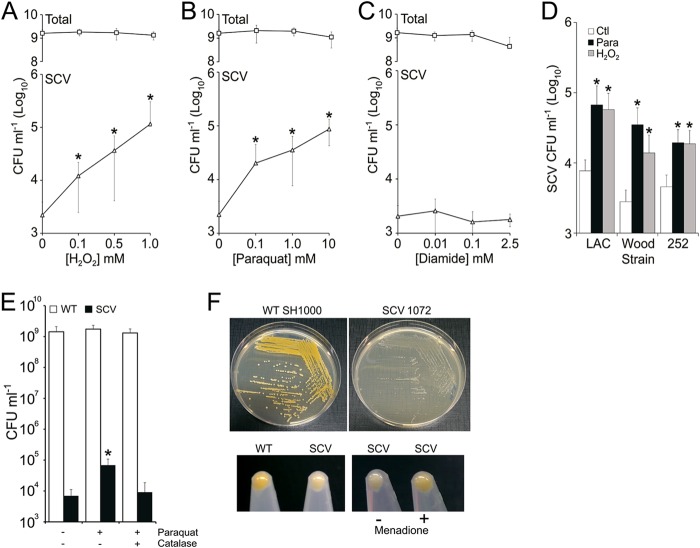
Hydrogen peroxide exposure leads to a specific, dose-dependent increase in the size of the SCV subpopulation. (A to C) S. aureus SH1000 was cultured in increasing concentrations of H_2_O_2_ (A), paraquat (B), or diamide (C) for 16 h, and the sizes of the total and SCV populations were determined. (D) The effect of H_2_O_2_ (1 mM) or paraquat (5 mM) on the size of the SCV subpopulations of strains USA300 LAC (LAC), Wood, and MRSA252 (252) were also determined. None of the oxidants used affected the size of the total population at the concentrations used (data not shown). (E) Wild-type S. aureus SH1000 was incubated in the absence (−) or presence (+) of paraquat and/or catalase, and the sizes of the total (open bars) and SCV populations (closed bars) were determined. (F) Colony morphology of wild-type S. aureus SH1000 (top left panel) and a representative menadione-auxotrophic SH1000-derived SCV (SCV1072) isolated from a culture containing paraquat (top right panel). The lack of pigment in the SCV seen on agar plates (top right panel) was also seen after liquid culture (bottom left panel). Culture of this SCV isolate in the presence of menadione restored pigmentation, indicating deficiencies in menaquinone production (bottom right panel) (2). Values which are significantly different (*P* < 0.05 [Student *t* test]) from oxidant-free conditions are indicated (*). These data represent the mean averages of 12 independent cultures. Error bars represent the standard deviations of the mean.

Increasing concentrations of H_2_O_2_ or paraquat, but not diamide, led to dose-dependent increases in the size of the gentamicin-resistant SCV subpopulation, which was up to 50-fold greater than in the absence of oxidative stress (Fig. 1A, B, and C). Similar effects of H_2_O_2_ (1 mM) and paraquat (5 mM) on the gentamicin-resistant SCV subpopulation were also observed for genetically diverse S. aureus strains USA300 LAC, Wood, and MRSA252 (Fig. 1D).

Paraquat generates superoxide radicals, which S. aureus can convert to H_2_O_2_ via superoxide dismutases (43). To determine whether the effect of paraquat on SCV numbers was due to H_2_O_2_ or superoxide production, S. aureus was cultured with paraquat in the presence or absence of purified bovine catalase (10 μg ml^−1^). The presence of exogenous catalase abrogated the effect of paraquat on SCV subpopulation expansion, indicating that H_2_O_2_ production, rather than superoxide, was responsible for the increased SCV frequency (Fig. 1E). However, it is possible that superoxide enhances H_2_O_2_-mediated damage by increasing free iron levels in the cell (54).

To investigate the nature of the recovered SCVs, representative colonies from independent cultures containing H_2_O_2_ or paraquat (*n* = 6) were examined, and each was found to have a typical SCV phenotype, with reduced pigmentation, and were classified as menadione auxotrophs (Fig. 1F). Whole-genome sequencing of a selection of each of the independently isolated menadione-auxotrophs (two from TSB only, three from TSB plus H_2_O_2_, and three from TSB plus paraquat) revealed mutations in genes in the menaquinone biosynthetic pathway (*aroB* and *menABDEF*) (Table 1) (55). These mutations were similar to those reported previously in clinical isolates and confirm that the SCV phenotype was due to genetic changes rather than to epigenetic effects or the physiological response of the bacterium to oxidative stress (15, 17).

### SCVs emerge during H_2_O_2_-induced bacteriostasis.

**FIG 2:**
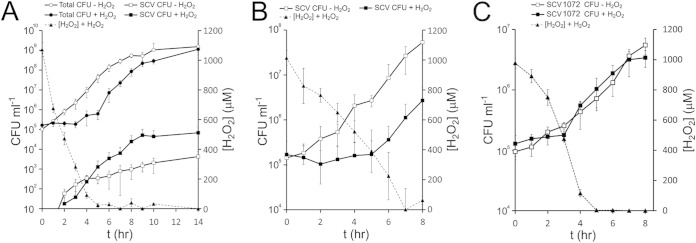
SCV emergence in S. aureus populations exposed to a bacteriostatic concentration of H_2_O_2_. (A) The numbers of wild-type (circles) and SCV (squares) CFU ml^−1^ were measured over time in the absence (open symbols) or presence (closed symbols) of 1 mM H_2_O_2_ (left axis). The concentration of H_2_O_2_ was also measured over time (dashed line, right axis). (B) The growth of a mixed population of phenotypically stable SCVs in the absence (open symbols) or presence (closed symbols) of H_2_O_2_ was also measured over time, together with the concentration of H_2_O_2_ (dashed line, axes as for panel A). (C) Growth of a single menadione-auxotrophic SCV (SCV1072) over time in the absence (open symbols) or presence (closed symbols) of H_2_O_2_. The concentration of H_2_O_2_ is indicated by the dashed line and the right-hand axis. The data points represent the mean average of 12 independent cultures. Error bars represent the standard deviations of the mean.

To understand how H_2_O_2_ modulates the size of the SCV subpopulation, we monitored the population dynamics of S. aureus during growth in the absence or presence of H_2_O_2_. As reported previously, wild-type S. aureus grew rapidly in the absence of H_2_O_2_ and produced a small SCV subpopulation during the early exponential phase (Fig. 2A) (22). In contrast, there was no change in total CFU counts in the presence of 1 mM H_2_O_2_, resulting in an extended lag phase that lasted until the H_2_O_2_ concentration was reduced to <400 μM (presumably due to the action of catalase and/or alkyl hydroperoxidase [40]). Once the H_2_O_2_ concentration was reduced, S. aureus replication began at a similar rate to that seen in the absence of H_2_O_2_ (Fig. 2A).

We hypothesized that the elevated rate of SCV emergence in the presence of H_2_O_2_ was either due to switching of wild-type bacteria into SCVs or, despite the small inoculum size, the replication of a very few SCVs present in the inoculum. To test whether SCVs could replicate in the presence of H_2_O_2_, we inoculated broth containing 1 mM H_2_O_2_ with a mixed population of phenotypically stable SCVs (including those auxotrophic for menadione, hemin, fatty acids, and those without identified auxotrophy) that represent the composition of SCVs found in cultures not exposed to oxidants and then monitored growth. Similar to wild-type bacteria, the growth of the SCV population was inhibited by 1 mM H_2_O_2_, leading to an extended lag phase relative to SCV growth in the absence of H_2_O_2_ (Fig. 2B). However, as seen for the wild-type population, once the concentration of H_2_O_2_ fell to ∼400 μM, SCV replication began (Fig. 2B). Because menadione-auxotrophic SCVs were the predominant SCV type isolated from cultures exposed to H_2_O_2_, we undertook a similar experiment to that described in Fig. 2B using a stable menadione auxotroph isolated from a culture exposed to paraquat (SCV1072). As for the wild-type and the mixed SCV inoculum, SCV1072 did not initiate replication until the H_2_O_2_ concentration had fallen to ∼400 μM (Fig. 2C). Therefore, SCV replication is not a viable explanation for the appearance of SCVs at early time points in cultures exposed to H_2_O_2_, when the oxidant is at concentrations inhibitory to staphylococcal growth.

### SCV emergence in the presence of H_2_O_2_ is dependent upon mutagenic DNA repair.

**FIG 3:**
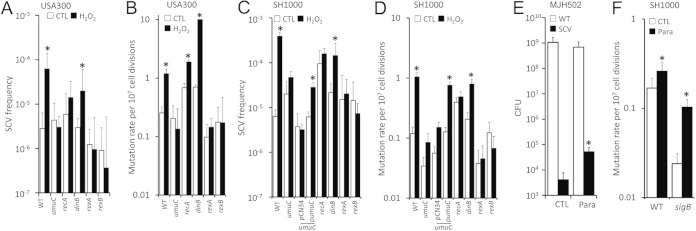
Expansion of the SCV subpopulation in response to H_2_O_2_ requires error-prone polymerase V under the control of the SOS regulon. (A) Frequency of SCVs in populations of WT S. aureus USA300 or transposon mutants lacking functional polymerase V (*umuC*::Tn), RecA (*recA*::Tn), polymerase IV (*dinB*::Tn), or RexAB (*rexA*::Tn and *rexB*::Tn) in the absence (open bars) or presence of H_2_O_2_ (filled bars). (B) Mutation rate of strains detailed in panel A grown in the absence (open bars) or presence (closed bars) of H_2_O_2_. (C and D) As for panels A and B but with strains constructed in the SH1000 background. In addition, panels C and D show data from the *umuC*::Tn mutant transformed with pCN34 only or pCN34 containing the *umuC* gene and promoter region (p*umuC*). (E) Total (WT) and SCV CFU counts from a SH1000-derived *sigB* mutant (MJH502) grown in the absence (CTL) or presence of paraquat (para). (F) Mutation rate of wild-type SH1000 (WT) and an SH1000-derived *sigB* mutant (*sigB*) grown in the absence (open bars) or presence (closed bars) of H_2_O_2_. The data in panels A, C, and E represent the mean averages of 12 independent cultures, and error bars represent the standard deviation of the mean. Values in panels B, D, and F represent the mutation rate as determined by fluctuation analysis, and error bars represent the 95% confidence intervals. Values which are significantly different (*P* < 0.05 [Student *t* test corrected for multiple comparisons via the Bonferroni method]) in the presence of H_2_O_2_ by comparison to those obtained in the absence of oxidants are indicated (*).

Because mutations have been shown to occur in stressed, nonreplicating E. coli cells via DNA double-strand break repair and the SOS response, which is strongly induced in S. aureus upon exposure to H_2_O_2_, we hypothesized that this may provide a mechanism for the emergence of SCVs under growth-inhibitory conditions (56–60). To test this, we utilized the NARSA transposon library to identify genes that were important for mutagenic DNA repair in the USA300 background (61). Wild-type and transposon mutants deficient in genes associated with DNA repair and the SOS response, including recombinase A (*recA*::Tn), error-prone polymerases IV or V (*dinB*::Tn, *umuC*::Tn), and *rexAB* (functionally equivalent to *recBCD* in E. coli), were grown in the absence or presence of H_2_O_2_. Several mutants, including *recA* and *rexAB* mutants, displayed increased sensitivity to H_2_O_2_, confirming a role in repair of damage caused by oxidative stress (data not shown). However, this increased sensitivity required a lower concentration of H_2_O_2_ (0.05 mM) to be used in these experiments compared to that of Fig. 1 and 2. Nonetheless, even at these reduced concentrations, H_2_O_2_ resulted in an increase in SCV frequency of ∼10-fold in wild-type S. aureus populations (Fig. 3A). In contrast, H_2_O_2_ exposure had no effect on the size of the SCV subpopulations of the *umuC*::Tn, *recA*::Tn, *rexA*::Tn, or *rexB*::Tn mutants relative to cultures without oxidant (Fig. 3A), indicating that double-strand break repair and the SOS response is required for SCV emergence during H_2_O_2_ exposure but not in its absence. In contrast, the mutant lacking functional *dinB* (which is not part of the S. aureus SOS regulon [62]) had only a slight defect in H_2_O_2_-induced SCV formation (Fig. 3A).

These findings were concordant with measurements of the mutation rate in S. aureus grown with or without H_2_O_2_, which showed that H_2_O_2_ exposure increased the mutation rate >5-fold in wild-type S. aureus but had no effect on the mutation rate of strains defective for *rexAB* or polymerase V (Fig. 3B). There was a modest (<3-fold) increase in the mutation rate of the *recA*::Tn mutant, but this was still significantly reduced compared to the wild-type (Fig. 3B). In contrast, there was no decrease in H_2_O_2_-induced mutation in S. aureus lacking polymerase IV (*dinB*) (Fig. 3B).

To ensure that these findings also applied to the SH1000 genetic background, DNA from *recA*::Tn, *rexA*::Tn *rexB*::Tn, *dinB*::Tn, and *umuC*::Tn was transduced into SH1000. Each of the DNA repair mutants behaved in a very similar manner to that described above for the USA300 mutants. Specifically, the mutants were defective for H_2_O_2_-induced SCV formation or mutation, with the exception of *dinB*::Tn (Fig. 3C and D). Complementation of the *umuC* coding sequence, under the control of the native promoter, to the *umuC*::Tn mutant restored H_2_O_2_-induced mutation and SCV formation, while the *umuC*::Tn mutant transformed with vector alone was defective for H_2_O_2_-induced mutation and SCV formation (Fig. 3C and D).

In E. coli, stress-induced mutation requires both the SOS response and a second signal via the RpoS sigma factor, which is part of the general stress response (63). Therefore, we considered the possibility that this may also be the case in S. aureus, especially since previous work has indicated that the alternative sigma factor SigB is required for SCV emergence in the presence of antibiotics (64, 65). However, the absence of SigB did not prevent an SCV population increase in the presence of 0.1 mM paraquat (Fig. 3E), and the *sigB* mutant was not defective for a paraquat-induced increase in the mutation rate (Fig. 3F). Therefore, SigB does not appear to be required for oxidative-stress-induced mutation in S. aureus. It should be noted, however, that we were unable to test higher concentrations of paraquat or H_2_O_2_ at any concentration used in Fig. 1 due to the increased sensitivity of the *sigB* mutant to oxidative stress (66). Taken together, these data demonstrate that components of the SOS response trigger switching from the wild-type to SCV phenotype via mutagenic DNA repair, which explains the emergence of SCVs in the presence of H_2_O_2_.

### H_2_O_2_ selects for phenotypically stable SCVs, enhancing population expansion via replication.

The data presented in Fig. 2A demonstrate that the SCV subpopulation emerged in the presence of H_2_O_2_ and continued to expand after the concentration of the oxidant fell below the growth-inhibitory concentration. However, it was not clear whether SCV population expansion at growth-permissive concentrations of H_2_O_2_ was predominantly due to the replication of a few SCVs generated by mutagenic DNA repair or was due to a very high rate of phenotype-switching from the wild-type to the SCV phenotype.

To investigate this, culture medium with or without oxidants was inoculated with ∼10^5^ CFU tetracycline-sensitive (Tet^s^) wild-type S. aureus ml^−1^ and ∼10 CFU SCVs from tetracycline-resistant SH1000 (Tet^r^) ml^−1^ and grown for 24 h. It should be noted that the mixed SCV subpopulation arose in cultures that had not been exposed to oxidants.

**FIG 4:**
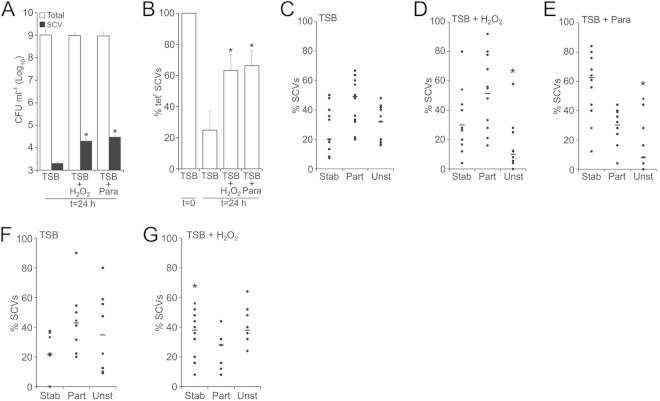
Hydrogen peroxide selects for the SCV phenotype. (A) Tryptic soy broth aliquots were inoculated with 10^5^ CFU tetracycline-sensitive wild-type S. aureus SH1000 and 10 CFU tetracycline-resistant SCVs in the absence (TSB) or presence of either H_2_O_2_ or paraquat (para), and the sizes of the total (open bars) and SCV (filled bars) populations were determined after 16 h of culture. (B) After 16 h of culture, the percentage of SCVs that were resistant to tetracycline was determined. Values significantly (*P* < 0.05 [Student *t* test]) different from those obtained with bacteria cultured in the absence of oxidants (TSB) are indicated (*). Bars represent the mean averages of 12 independent cultures. Error bars represent the standard deviations of the mean. (C, D, and E) The relative stability of SCVs isolated from cultures without (TSB) (C) or with H_2_O_2_ (D) or paraquat (para) (E) were determined by using a previously described assay (22). Individual SCVs were classified as stable (stab), partially stable (part), or unstable (unst) as described in Materials and Methods. The data points represent a single independent culture. The percentag of SCVs classified as unstable was significantly lower in cultures containing oxidants than those without (an asterisk [*] indicates a significant difference relative to TSB without oxidants). (F and G) SCVs that were incubated either in the absence (F) or presence (G) of a subinhibitory concentration of H_2_O_2_ were assessed for phenotypic stability. Each data point represents a single independent culture. SCVs that were exposed to H_2_O_2_ or paraquat were significantly more stable than those incubated in TSB alone. Each data point represents a single culture (10 from each condition). Significant differences between each stability category (*P* < 0.05 [Student *t* test corrected for multiple comparisons via the Bonferroni method]) are indicated (*).

As expected, the size of the SCV subpopulation in cultures containing oxidants was greater than those without oxidants (Fig. 4A). In the absence of oxidative stress, the percentage of SCVs that were tetracycline resistant fell from 100% in the inoculum to ca. 20% in the mature culture, indicating that 80% of the final SCV subpopulation had arisen via phenotype switching from the tetracycline-sensitive wild-type population (Fig. 4B), i.e., SCVs revert at high frequency in the absence of oxidative stress. In contrast, in the presence of H_2_O_2_ or paraquat, the percentage of tetracycline-resistant SCVs at 24 h were ca. 60% each, indicating that these oxidative stresses select for maintenance of the SCV phenotype and that SCV reversion to the wild type does not occur at a high frequency in the presence of oxidants (Fig. 4B).

It has been shown previously that SCV replication is associated with an increase in phenotypic stability, since unstable SCVs revert to the wild-type (22). To test whether oxidants select for SCV stability, we used a previously described stability assay (22) and found that SCVs that arose in cultures exposed to H_2_O_2_ or paraquat were significantly more stable than those that arose in broth only (Fig. 4C, D, and E). We then generated a pool of SCVs that had arisen spontaneously in cultures not exposed to oxidants and then grew them in the absence or presence of H_2_O_2_. Oxidative stress resulted in significantly increased SCV stability, demonstrating that H_2_O_2_ selects for SCV stability regardless of whether SCVs arose via the SOS response or spontaneously (Fig. 4F and G). Therefore, the exposure of SCVs to oxidants results in enhanced stability, which reduces reversion to the wild-type and thus enables SCV population expansion via replication.

### Nonoxidative SOS induction promotes SCV reversion to the wild type.

The data presented in Fig. 4 strongly suggest that H_2_O_2_ selects for phenotypically stable SCVs, which was surprising because activation of the SOS response would be expected to increase the frequency of suppressor mutations which promote SCV reversion to the wild-type phenotype. This suggests that the selective pressure exerted by oxidants on SCVs is great enough to overcome the increased mutation rate caused by induction of the SOS response.

However, we considered two alternative explanations for the enhanced stability of SCVs exposed to oxidative stress: that SCVs generated by the SOS response are inherently more stable than those that arise spontaneously or that the SOS response cannot trigger reversion of SCVs to wild-type bacteria.

**FIG 5:**
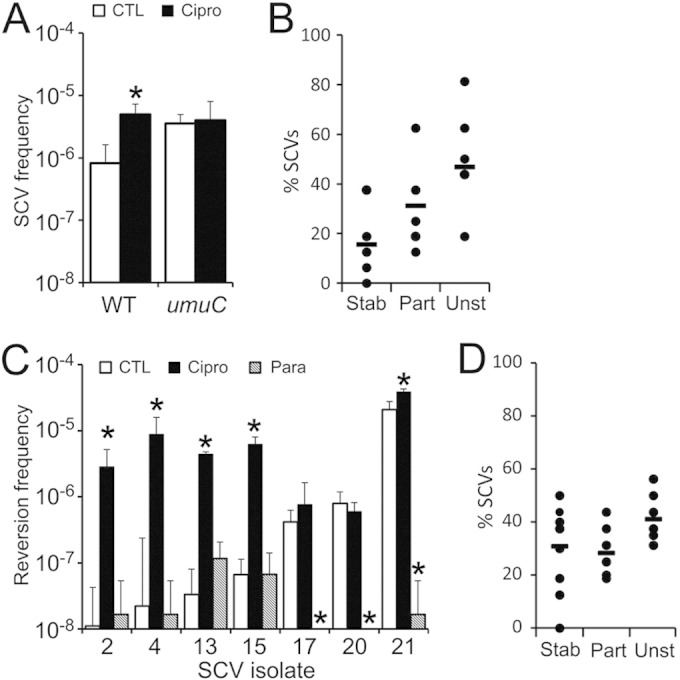
Ciprofloxacin promotes bidirectional phenotype-switching. (A) Wild-type SH1000 and a *umuC* mutant were grown in the absence (□) or presence (■) of a subinhibitory concentration of ciprofloxacin for 16 h, and the frequency of gentamicin-resistant SCVs was determined. The data represent the mean average of 10 independent cultures, and error bars represent the standard deviations. Values which differ from those seen in media lacking ciprofloxacin are highlighted (*). (B) Stability of SCV isolates that arose in wild-type SH1000 populations in the presence of ciprofloxacin (*n* = 6). The data are presented and analyzed as described in the legend to Fig. 4. (C) The frequency of SCV reversion to the wild type was determined in the absence (CTL) or presence of ciprofloxacin (Cipro) or paraquat (Para). Reversion rates that differ from the those found on media without supplements are highlighted (*). (D) Stability of SCVs that arose in the *umuC* background in the absence of oxidants or ciprofloxacin (*n* = 8).

To test these possibilities, we used the antibiotic ciprofloxacin, which induces a very similar DNA damage repair to that described upon H_2_O_2_ exposure (56, 62). Exposure of wild-type but not *umuC*::Tn mutant bacteria to a subinhibitory concentration of ciprofloxacin led to an increase in SCV frequency, confirming that induction of the SOS response promotes SCV emergence via mutagenic DNA repair (Fig. 5A). However, the SCVs triggered by ciprofloxacin were no more stable than those which emerged in the absence of the antibiotic (Fig. 4C and 5B). This demonstrates that SCVs generated via the SOS response are not inherently more stable than those that arise spontaneously during bacterial replication. We then tested whether the SOS response can promote SCV reversion to the wild type by exposing a panel of SCVs with various levels of stability to ciprofloxacin or paraquat. In five of the seven SCVs examined, ciprofloxacin exposure promoted the frequency of reversions, indicating that SOS induction can indeed promote SCV reversion to the wild type (Fig. 5C). In contrast, exposure of each of the SCVs to paraquat using the same assay either had no effect or reduced SCV reversion frequency (Fig. 5C). Therefore, while both ciprofloxacin and paraquat stress trigger the SOS mutagenic repair pathway, only the antibiotic promotes bidirectional switching between the wild type and SCVs. In contrast, oxidative stress triggers wild-type-to-SCV switching but selects against SCV reversion to the wild type.

Finally, we examined whether loss of mutagenic DNA repair affected SCV stability in the absence of SOS-inducing stresses. This revealed that SCVs generated by the *umuC*::Tn mutant in the SH1000 background were as stable as those that arise in the wild type, demonstrating that the SOS response does not play a role in SCV emergence or reversion in the absence of genotoxic stresses (Fig. 4C and 5D).

### SCVs are less susceptible to H_2_O_2_ than parental strains.

**FIG 6:**
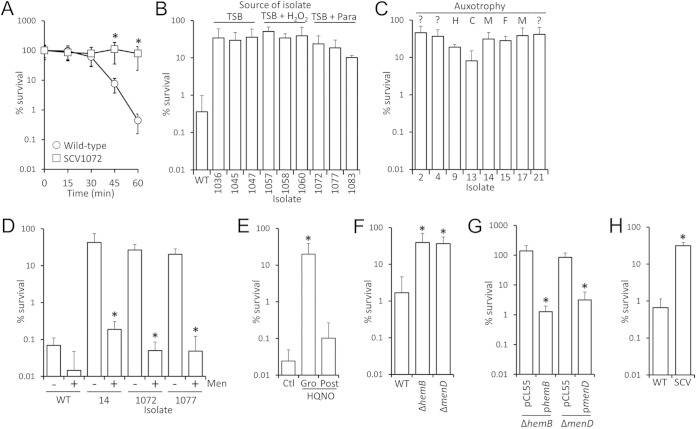
SCVs are more resistant to H_2_O_2_ than wild-type bacteria. (A) The survival of wild-type SH1000 S. aureus (○) or a derived SCV isolate (□) during exposure to 30 mM H_2_O_2_ was determined by quantifying CFU. Survival of the SCV was significantly greater (*P* < 0.05) than that of the wild type at the 45- and 60-min time points. (B) Survival of three independently isolated, menadione-auxotrophic SCVs from cultures without (TSB) or with H_2_O_2_ or paraquat (para) was determined after 60 min of exposure to 30 mM H_2_O_2_. The survival of the wild type after 60 min is shown for comparison. The survival of all SCV isolates was significantly greater than that of the wild type. (C) Survival of independently isolated SCVs with auxotrophy for hemin (H), CO_2_ (C), menadione (M), fatty acids (F), or where auxotrophy has not been established (?) after 60 min in 30 mM H_2_O_2_. (D) Survival of wild-type or various, independently isolated menadione-auxotrophic SCVs from cultures without oxidants, grown in the absence (−) or presence (+) of menadione. Supplementation of SCV but not wild-type cultures with menadione significantly reduced survival in the presence of H_2_O_2_. (E) Growth of S. aureus SH1000 in the presence of the electron-transport chain inhibitor HQNO (Gro) promotes resistance to H_2_O_2_ relative to growth in TSB only (Ctl) or growth in TSB, followed by addition of HQNO to bacteria 5 min prior to H_2_O_2_ exposure (Post). (F) Survival of the USA300 wild-type strain and derived deletion mutants lacking *hemB* or *menD* after incubation in 25 mM H_2_O_2_. Survival of the SCVs was significantly greater than WT. (G) Survival after incubation in 25 mM H_2_O_2_ of USA300 *hemB* and *menD* mutant strains transformed either with pCL55 or PCL55 containing the *hemB* (p*hemB*) or *menD* (p*menD*) coding sequences. Survival of complemented strains was significantly lower than that of mutants transformed with vector alone (pCL55). (H) Survival of a clinical menadione-auxotrophic SCV (CX003SCV) and derived revertant (CX003WT) with the wild-type phenotype after incubation in 30 mM H_2_O_2_. Survival of the SCV was significantly greater than wild-type after 60 min. Significance was determined by using a Student *t* test corrected for multiple comparisons via the Bonferroni method and declared significant when *P* < 0.05.

Previous work has suggested that SCVs should be more susceptible to H_2_O_2_ than wild-type bacteria due to the lack of staphyloxanthin pigment and reduced catalase activity in heme auxotrophs (19, 36, 37, 45). However, since H_2_O_2_ selected for the SCV phenotype we considered the possibility that SCVs are in fact less sensitive to oxidative stress than wild-type bacteria. To test this, the survival of wild-type SH1000 in the presence of 30 mM H_2_O_2_ was compared to a phenotypically stable SCV isolate that arose in the presence of paraquat (SCV1072). This revealed that survival of the SCV was significantly greater than that of the wild type (Fig. 6A). Further analyses of three stable menadione-auxotrophic SCVs from independent cultures containing either H_2_O_2_ or paraquat revealed that each SCV isolate was significantly more resistant to H_2_O_2_ killing than the WT strain (Fig. 6B). Because culture in the presence of H_2_O_2_ or paraquat may have selected for mutations that confer elevated resistance to oxidative stress, three additional, independently isolated menadione-auxotrophic SCVs, which arose in broth without oxidants, were assessed. These showed similarly high levels of resistance to H_2_O_2_ killing (Fig. 6B), suggesting that H_2_O_2_ resistance is an intrinsic property of menadione-auxotrophic SCVs.

To determine whether resistance to H_2_O_2_ killing was related to the auxotrophic phenotype a panel of SCVs, isolated from gentamicin-containing media, with various or unknown auxotrophies was assessed for resistance to H_2_O_2_ killing. All of these isolates were significantly more resistant to H_2_O_2_ killing than the wild-type strain (Fig. 6C).

We also considered the possibility that gentamicin-resistant SCVs may consistently accumulate mutations which decrease susceptibility to H_2_O_2_. To test this, menadione-auxotrophic SCV isolates were cultured in the absence or presence of menadione, and their susceptibility to H_2_O_2_ killing was determined. Culture of menadione-auxotrophic SCVs in the presence of menadione produced bacteria that were as sensitive as the wild-type parental strain to H_2_O_2_, indicating that secondary mutations are not responsible for the elevated H_2_O_2_ resistance (Fig. 6D). It should be noted that menadione has been used as a redox cycling agent to generate superoxide within bacteria. However, the concentrations typically used in such studies are ∼1,000-fold greater than those used here, and there was no significant effect on the viability of the wild-type bacteria in the presence of H_2_O_2_ (2, 67). To further test whether reduced susceptibility to H_2_O_2_ was solely due to defects in the electron transport chain, S. aureus wild type was cultured in the presence of the Pseudomonas exoproduct HQNO, which blocks the electron transport chain of Gram-positive bacteria and confers an SCV phenotype upon S. aureus (68). Culture of S. aureus in the presence of HQNO produced bacteria that were resistant to H_2_O_2_ killing (Fig. 6E). However, the presence of HQNO alone did not alter H_2_O_2_ resistance of S. aureus which had been cultured in the absence of the exoproduct (Fig. 6E). Therefore, simply blocking the electron transport chain is not protective against H_2_O_2_. Rather, resistance is most likely due to the physiological adaptation of S. aureus to loss of the electron transport chain.

To ensure that these findings were of clinical relevance, we assessed the survival of *hemB* and *menD* deletion mutants constructed in the USA300 community-associated MRSA strain. In keeping with the data for SH1000, the survival of wild-type USA300 in the presence of H_2_O_2_ was significantly lower than that of isogenic *hemB* or *menD* mutants (Fig. 6F). Complementation of either mutant with the relevant coding sequence restored the wild-type growth phenotype (data not shown) and resulted in decreased survival in H_2_O_2_, whereas vector alone did not affect growth or survival (Fig. 6G). Finally, increased H_2_O_2_ resistance was demonstrated in a clinical menadione-auxotrophic SCV isolate (CX003SCV), relative to a wild-type revertant (CX003WT) (Fig. 6H). Therefore, resistance to H_2_O_2_ is an inherent property of electron-transport chain-deficient SCVs that very likely contributes to their ability to persist within host tissues during chronic infections.

### Elevated catalase activity in SCVs partially explains enhanced H_2_O_2_ resistance.

In addition to staphyloxanthin, catalase is a major staphylococcal defense against H_2_O_2_ and has been reported to be expressed at higher levels in clinical SCVs than wild-type S. aureus (33, 40–42). However, heme-auxotrophic SCVs cannot generate functional catalase and SCVs isolated from the lungs of patients with cystic fibrosis have been reported to have reduced catalase activity (5, 45).

**FIG 7:**
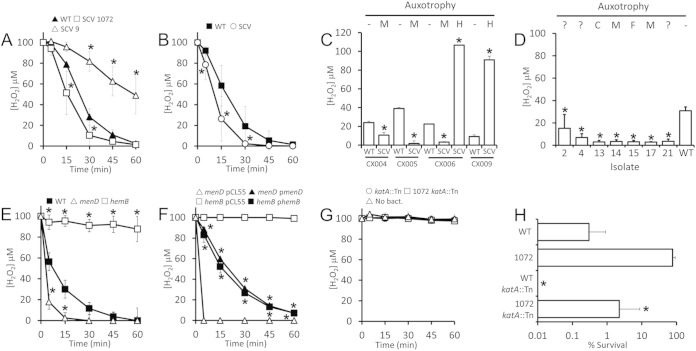
Most SCVs display elevated catalase production, which partially explains their decreased susceptibility to H_2_O_2_. (A) The H_2_O_2_ concentration was measured over time during incubation with SH1000 wild type, menadione-auxotrophic SCV1072 (1072) or heme-auxotrophic SCV9 (9). (B) H_2_O_2_ degradation by a clinical menadione-auxotrophic SCV isolate (SCV) and a revertant with wild-type (WT) phenotype. (C) H_2_O_2_ degradation after 30 min of incubation by clinical SCV isolates auxotrophic for menadione (M) or hemin (H) and paired revertant isolates. (D) H_2_O_2_ degradation after 30 min of incubation by SH1000-derived SCV isolates with diverse or unidentified auxotrophies, including CO_2_ (C), menadione (M), fatty acids (F), or where auxotrophy has not been established (?) or does not exist. Wild-type (WT) S. aureus SH1000 is included as a control. (E) H_2_O_2_ degradation by wild-type USA300 and *menD* (*menD*) and *hemB* (*hemB*) mutants. (F) H_2_O_2_ degradation by *menD* (*menD*) and *hemB* (*hemB*) mutants transformed with plasmids containing the deleted genes (p*menD* or p*hemB*) or vector only (pCL55). (G) H_2_O_2_ levels after incubation with SH1000 *katA*::Tn (*katA*::Tn) or SCV1072 *katA*::Tn (1072 *katA*::Tn) or in the absence of bacteria (No bact.). (H) Survival of wild-type (WT) SH1000, menadione-auxotrophic SCV1072 (1072), SH1000 *katA*::Tn (*katA*::Tn), or SCV1072 *katA*::Tn (1072 *katA*::Tn) after incubation in 30 mM H_2_O_2_ for 1 h. All data points represent the means of four independent experiments performed in duplicate. Error bars represent the standard deviations of the mean. Values that are significantly different from the wild type are denoted (*), with the exception of panel F, in which a significant difference from strains expressing catalase is denoted. Significance was determined by using a Student *t* test corrected for multiple comparisons via the Bonferroni method and declared significant when *P* < 0.05.

To resolve the question of catalase activity in electron-transport chain-deficient SCVs, we measured the ability of wild-type S. aureus SH1000, menadione-auxotrophic SCV isolate SCV1072 and heme-auxotrophic SCV9 to degrade H_2_O_2_. This revealed significantly elevated catalase activity in the menadione-auxotrophic SCV relative to the wild type, while the catalase activity of the heme-auxotrophic SCV was significantly impaired relative to wild type (Fig. 7A). Similarly, a clinical menadione-auxotrophic SCV isolate had significantly higher catalase activity than a revertant isolate with the wild-type phenotype (Fig. 7B). In keeping with these data, analyses of an additional four clinical SCV isolates and matching revertants revealed that menadione-auxotrophic SCVs degraded significantly more H_2_O_2_ than revertants, whereas heme auxotrophs exhibited defective catalase activity (Fig. 7C).

Next, we examined catalase activity in a panel of gentamicin-resistant SCVs with diverse auxotrophies or no identified auxotrophy isolated from broth cultures in the absence of oxidants. In every case, the catalase activity of the SCV was greater than that of the wild type, although significant variation was observed between isolates (Fig. 7D). Finally, to demonstrate that selection for SCVs does not itself select for elevated catalase activity, we measured catalase in isogenic *menD* and *hemB* mutants and the wild-type USA300 parent strain. Consistent with the previous data, this revealed that the *menD* deletion mutant had significantly higher levels of catalase activity than wild-type USA300, while the *hemB* deletion mutant was unable to degrade H_2_O_2_ (Fig. 7E) (45). Complementation of the *hemB* and *menD* mutants with the relevant wild-type coding sequence restored catalase activity to wild-type levels (Fig. 7F).

To confirm that the degradation of H_2_O_2_ was due to catalase, rather than alkyl-hydroperoxidase or other peroxidases, we transduced SH1000 WT and SCV1072 with DNA from a USA300 *katA*::Tn mutant (40). Both strains were completely devoid of catalase activity, confirming the role of catalase (KatA) in the H_2_O_2_ breakdown (Fig. 7G).

Next, we sought to determine whether catalase activity explained the enhanced resistance of menadione-auxotrophic SCVs to H_2_O_2_ (Fig. 6A and B). Wild-type SH1000, SCV1072, SH1000 *katA*::Tn, and SCV1072 *katA*::Tn were each exposed to 30 mM H_2_O_2_ for 1 h, and the survival was determined. Strains deficient in catalase showed increased sensitivity to H_2_O_2_ (Fig. 7H). However, SCV1072 *katA*::Tn was not as sensitive to H_2_O_2_ as SH1000 *katA*::Tn, indicating that elevated catalase activity only partially explains the resistance of menadione-auxotrophic SCVs to H_2_O_2_ (Fig. 7H).

Taken together, these data indicate that enhanced catalase activity is common to most electron transport chain-deficient SCVs, with the exception of those that cannot synthesize heme. However, additional factors beyond catalase contribute to the resistance of SCVs to H_2_O_2_, particularly in heme auxotrophs.

## DISCUSSION

S. aureus is responsible for a raft of chronic and recurrent infections despite triggering a potent immune response and antibiotic therapy (69, 70). During the course of infection, S. aureus frequently acquires mutations which promote survival in host tissues, including those that confer a small-colony variant phenotype. The data presented in this report reveal that these mutations increase in frequency in response to one of the major ROS produced by neutrophils, H_2_O_2_, via the SOS response. These data support previous work showing that increases in the mutation rate following DNA damage are due to the action of specific repair machinery, rather than the DNA damage itself (57–60).

The ability of bacteria to transiently increase mutation rates in response to environmental stress increases the probability of beneficial (adaptive) mutations that enhance survival (58, 71). Certainly, the emergence of electron transport chain-deficient SCVs in response to oxidative stress appears to be beneficial to S. aureus due to their resistance to oxidative stress and enhanced catalase production, which may enhance survival and/or replication of wild-type bacteria via detoxification of H_2_O_2_ (Fig. 6 and 7). In addition, SCVs have a number of other phenotypic properties which might promote persistence in host tissues, including intracellular survival, strong biofilm formation, and a high degree of antibiotic tolerance (5, 12, 13, 30–32). Therefore, a single inactivating mutation in the menaquinone biosynthetic pathway has a profound effect on the phenotype of S. aureus, changing it from a fast-growing, toxin-producing pathogen to a much less pathogenic and slow-growing variant that is able to persist within host tissues for extended periods. However, the close correlation between mutation rate (as determined by mutations at the *rpoB* locus) and SCV emergence indicates that the *men* operon is probably not a mutation hot spot, at least with respect to H_2_O_2_-associated mutations.

In E. coli, stress-induced mutation involves the low-fidelity polymerases IV and V. Although the ability of a bacterium to increase the mutation rate is a beneficial tool, the principal function of these polymerases is the replication of damaged DNA in a process known as “trans-lesion synthesis,” the low-fidelity nature of the polymerase enabling it to bypass DNA lesions at the cost of a high-frequency of base pair mismatches (72). However, it is not clear whether the increased mutation rate associated with polymerases IV and V is simply a consequence of DNA repair or part of a coevolved mechanism to promote the mutation rate during times of DNA-damaging stress and thus increase the likelihood of beneficial mutations arising.

In S. aureus, trans-lesion synthesis appears to make a small contribution to S. aureus resistance to oxidative stress since the *umuC*::Tn mutant lacking polymerase V (but not *dinB/*polymerase IV) was slightly more sensitive to H_2_O_2_ than the wild type (data not shown). These data fit with previous work which shows that the expression of *umuC*, but not *dinB*, is increased in response to H_2_O_2_ (56). Therefore, in S. aureus, the expression of polymerase V appears to facilitate efficient repair of H_2_O_2_-mediated DNA damage. However, this does not rule out the possibility that polymerase V is part of a coevolved mechanism to increase the mutation rate in response to environmental stress. For example, *umuC* is one of the most strongly expressed genes in response to various genotoxic stresses, and this may result in greater polymerase V production than is strictly necessary to repair the damaged DNA (56, 62, 73).

Although H_2_O_2_ exposure led to large increases in SCV frequency, this was not solely due to an elevated mutation rate but also to the subsequent replication of emergent SCVs. H_2_O_2_ selected for the SCV phenotype, which may reflect the enhanced resistance of SCVs to H_2_O_2_, coupled with enhanced catalase production. Therefore, with the possible exception of heme auxotrophs, which lack catalase activity, gentamicin-resistant SCVs appear to be well equipped to persist in environments with a high burden of ROS. This correlates with the clinical evidence that SCVs are able to persist in host tissues, resisting clearance by immune cells that expose the pathogen to the oxidative burst (1–14, 36, 37).

The discovery of enhanced catalase activity in non-heme-auxotrophic SCVs is in keeping with a transcriptomic study of clinical SCV isolates, which reported enhanced *katA* expression (33). Also in keeping with previous work, proteomics analysis of clinical and *in vitro* selected heme-auxotrophic SCVS revealed reduced catalase than in corresponding wild-type bacteria (74). Therefore, it appears that loss of the electron transport chain results in enhanced expression of *katA*, leading to elevated catalase activity, except where heme biosynthesis is defective (33, 74). The reason why catalase activity is elevated in menadione auxotrophic SCVs is under investigation but may reflect the significantly altered metabolic profile of these mutants, which results in altered production of virulence factors and defense molecules such as staphyloxanthin (discussed below) (8, 9, 14, 19, 28, 30, 36, 45). It could, therefore, be hypothesized that enhanced catalase activity is a compensatory mechanism for the loss of staphyloxanthin, but this remains to be tested.

Although there appear to be a number of different pathways by which electron transport chain-deficient SCVs can arise (resulting in diverse auxotrophies), cultures exposed to H_2_O_2_ consistently generated menadione auxotrophs. Since menadione auxotrophs were no more resistant to H_2_O_2_ and produced similar levels of catalase to other SCVs (with the exception of heme auxotrophs), this is most likely explained by the increased likelihood of this variant arising relative to others. Specifically, menadione-auxotrophic SCVs can arise via inactivating mutations anywhere in the menaquinone biosynthetic pathway, whereas other types of SCV might only arise via mutations in much smaller loci. In support of this hypothesis, in cultures not exposed to H_2_O_2_, menadione-auxotrophic SCVs were the most abundant (40%), followed by heme auxotrophs (35%). Therefore, it appears that H_2_O_2_ selects for catalase producing SCVs, of which menadione auxotrophs are the most abundant, over the catalase-deficient heme auxotroph.

While SCVs are resistant to oxidative stress and have many phenotypic properties which promote survival in host tissues, these come at the cost of slow growth and loss of exotoxin production (2, 4, 6, 9, 13, 35). Therefore, S. aureus populations must provide a balance between fast-growing, toxin-producing wild-type bacteria which are essential for the establishment of infection and slow-growing non-toxin-producing SCVs, which are able to resist threats such as oxidative stress or antibiotics. Indeed, such a strategy parallels the formation of antibiotic tolerant persister cells (75, 76). Balaban et al. showed that persister cells arise stochastically during growth (type I) and that the frequency increases in response to specific environmental stresses such as subinhibitory concentrations of antibiotics (type II) (75, 77). The production of persister cells prior to antibiotic exposure is hypothesized to be a bet-hedging strategy to ensure the population against exposure to lethal concentrations of antimicrobials that would otherwise eradicate the entire population (75–79).

Although SCVs arise via mutation and persister cells via changes in the physiological state of cells, both events are stochastic in nature and the frequency of these events is influenced by genetic factors and there are, therefore, clear parallels in their emergence within populations (77). We have previously shown that SCVs emerge constitutively in replicating S. aureus cultures (type I) and in this report demonstrate that a specific environmental stress enhances SCV emergence and population size via the action of specific gene products (type II) (22). Therefore, we hypothesize that SCVs comprise a bet-hedging strategy against lethal oxidative and antibiotic stress in a similar way to persisters ensuring populations against bactericidal antibiotics. A key part of such an ensurance policy is the ability to restore the population of wild-type bacteria, which SCVs can do via the repair of mutations or acquisition of suppressor mutations that restore the function of mutated gene products (15, 17). In addition, activation of the SOS mutagenic repair pathway via subinhibitory ciprofloxacin (but not oxidative stress) can promote SCV reversion to the wild type.

The very high resistance of SCVs to concentrations of H_2_O_2_ that are lethal to the wild type was a surprising finding given the reduced pigmentation (and catalase levels in the hemin auxotroph). The SCVs that arose under oxidative stress were gentamicin resistant and consistently auxotrophic for menadione, indicating loss of menaquinone biosynthesis and thus interruption of the electron transport chain (2, 9).

The ability of electron transport chain-deficient bacteria to resist H_2_O_2_ is in apparent contrast to previous work which showed that blockage of the electron transport chain of E. coli using KCN, or disruption of the *menA* gene, resulted in increased susceptibility to H_2_O_2_ (80). Loss of the electron transport chain in E. coli led to a significant increase in reducing power inside the cell, which propagates the highly damaging Fenton reaction by reducing iron (80). Our experiments with HQNO demonstrate that SCV resistance to H_2_O_2_ is not simply a function of a defective electron transport chain. Rather, it is only when S. aureus has been cultured in the absence of a functional electron transport chain that it is able to survive subsequent H_2_O_2_ challenge. Although this is partially due to catalase activity, additional factors promote the resistance of electron transport chain-deficient S. aureus to H_2_O_2_. For example, S. aureus can avoid redox stress during loss of the electron transport chain by switching to fermentative metabolism via the redox-regulatory element Rex (81–83). Metabolic and transcriptomic analyses of SCVs reveal a huge increase in lactate and alcohol dehydrogenase activity, and this maintains redox balance in the cell, preventing an accumulation of reducing power (14, 30, 83). Furthermore, it is possible that fermentative metabolism renders SCVs more resistant to H_2_O_2_ killing by reducing the need for iron-containing metabolic enzymes in the cytoplasm, as well as cytochromes. In support of this hypothesis, wild-type S. aureus exposed to H_2_O_2_ increase expression of genes associated with fermentation and a Staphylococcus epidermidis mutant lacking a functional TCA cycle displayed elevated resistance to H_2_O_2_ killing (54, 55, 84).

Taken together, the data presented here reveal an additional strategy by which S. aureus can promote its survival under conditions of oxidative stress via the production of small-colony variants in response to H_2_O_2_ exposure. In addition to ensuring the population against potentially lethal oxidative stress, elevated SCV production is likely to promote persistent infection via reduced susceptibility to antibiotic therapy, increased biofilm formation, and enhanced intracellular persistence.
